# Effect of Head Position Change on Endotracheal Cuff Pressure in Mechanically Ventilated Patients: A Quasi-Experimental Study

**Published:** 2020-11

**Authors:** Roghieh Nazari, Mohammad Salehpour Omran, Hamid Sharif Nia, Ameneh Yaghoobzadeh

**Affiliations:** 1 Amol Faculty of Nursing and Midwifery, Mazandaran University of Medical Sciences, Sari, Iran,; 2 Student Research Committee, Mazandaran University of Medical Sciences, Sari, Iran,; 3 Department of Geriatric Nursing, School of Nursing and Midwifery, Tehran University of Medical Sciences, Tehran, Iran

**Keywords:** Endotracheal Intubation, Cuff pressure, Head position, Intensive Care Unit

## Abstract

**Background:**

Endotracheal cuff pressure may be affected by various factors and interventions. Therefore, this study was conducted to investigate the effect of head position change on cuff pressure of the endotracheal tube whilst undergoing mechanical ventilation.

**Materials and Methods:**

In this semi-experimental study, 61 intubated patients undergoing mechanical ventilation were studied. Each subject was used as their own control group. First, each patient was placed in starting position and the cuff pressure was adjusted in the range of 20–30 cmH_2_O. Then, the head position was placed in anterior flexion, hyperextension, left lateral flexion, right lateral flexion, left rotation, and right rotation without separating the manometer from the pilot cuff. The cuff pressure was recorded and analyzed at each stage.

**Results:**

The endotracheal cuff pressure increased in all six head positions. The highest difference in pressure was observed in the anterior flexion and left rotation positions (p<0.001). The observed increases in cuff pressure were higher than the normal range (30 cmH_2_O) in a significant number of patients.

**Conclusion:**

Changing the head position in mechanically ventilated patients increases endotracheal cuff pressure. Therefore, it is suggested that the cuff pressure should be re-examined and adjusted after each head position change while avoiding unnecessary movements of the head and neck of the intubated patients.

## INTRODUCTION

It is essential to use endotracheal tubes to provide a safe and secure airway in patients during surgery and mechanical ventilation (1). Although endotracheal tube with low-volume and low-pressure are still used, an important aspect of airway management in these patients is the care of the cuff and filling it with proper pressure (2). Failure to do so will cause complications; prolonged cuff pressure leads to subglottic ulcer, hoarseness of voice, nerve damage, damage to the tracheal wall, stenosis and tracheal fistula (3). Low cuff pressure due to micro-aspiration will cause the patient to suffer from ventilator associated pneumonia (VAP)(4). Therefore, it is necessary to measure the cuff pressure by measuring the pressure in the pilot balloon of the endotracheal tube cuff (5). The pressure should be set at greater than 20 cmH_2_o to prevent VAP (6), and should not exceed 30 cmH_2_O to minimize the risk of damage to the tracheal mucosa (2). Preserving the endotracheal cuff pressure in a desirable range is a challenging issue because cuff pressure may be influenced by various patient-related factors, environmental conditions, and nursing interventions(1).

A review of the literature showed that some factors such as positive pressure ventilation (7), bronchoconstriction, spasticity, edema (1), head flexion (8) and some health care interventions (9) can result in increased cuff pressures. On the other hand, others factors such as sedation, neuromuscular block, increase in central temperature (3), time passage and head extension (8) can reduce cuff pressure. Although changing the position of the patient, especially in the head and neck, is one of the common interventions in the intensive care unit (ICU) performed for different purposes and during different procedures, very little information has been found about how head position could affect the cuff pressure of the endotracheal tube. Therefore, more studies are needed in this regard to develop strategies to maintain cuff pressure in a safe range and to prevent relevant complications. Therefore, this study was conducted to investigate the effect of head position change on cuff pressure of the endotracheal tube whilst undergoing mechanical ventilation.

## MATERIALS AND METHODS

This study was performed using a pretest-posttest semi-experimental design and was registered with the Iranian Center for Clinical Trials (IRCT20110705006955N4). Subjects were recruited from the intubated patients admitted to the ICU of 17 Shahrivar Hospital of Amol. Based on the findings of the Lizy et al. study (1), the suitability of the sample size was calculated to be 61 participants using GPower, two tailed significance level (an alpha significant level of .05), β equal to 0.8, and the effect size of 0.36.

Convenient sampling method was used in selecting the patients. All patients with the following features were eligible for inclusion to the study: undergoing mechanical ventilation with oral endotracheal intubation, at least 18 years of age, having adequate sedation level (giving 5 scores using Richmond Agitation-Sedation Scale), being non-pregnant, having central temperature of 35–37.5 °C, body mass index (BMI) <30, no contradiction for changing the head position, high-frequency oscillatory ventilation, not being in the prone position, and not having difficult intubation.

The endotracheal tubes for all patients were made by the same company and their types were high volume and low pressure cuffs at distal end as well as pilot cuff with non-return valve. Each subject was used as his/her control in order to collect data. At first, each patient was placed in a supine position with a head position of 30 degree. A digital protractor was used to determine the bed angle. This position was considered as the starting state. In this position, the head was in a neutral position (i.e. without any rotation, flexion or extension). After placing the head in the starting position, the pilot balloon of the endotracheal tube was connected to the cuff pressure manometer. The cuff pressure was adjusted to be in the range of 20 to 30 cmH_2_O, and no air leak was confirmed. The air leakage around the cuff was controlled through hearing the cuff location. Then, the patients` head position was placed in six positions; anterior flexion, hyperextension, left lateral flexion, right lateral flexion, left rotation, and right rotation. These positions were chosen because they are part of the routine care of mechanically ventilated patients. Positional changes were performed sequentially and the patient was not returned to the starting position after each measurement. At least five minutes passed between each head change position. The end-expiratory ventilator key was held for about 4 to 5 seconds when the patient was placed in each of the positions. At the same time, the cuff pressure was measured and recorded. Care was taken to avoid any traction in the trachea and no additional maneuvers were performed during this procedure. Subjects were excluded if they required any intervention such as suction. Moreover, any resistance or reaction from the patient to any positional changes resulted in ceasing the test and exclusion from the study. Since the patients had sufficient level of sedation, they did not have any resistance or reaction to the changing position so no patients were excluded for this reason.

The CONSORT flow diagram is shown in Fig. 1 The normal distribution of data was estimated using the Kolmogorov-Smirnov test in order to analyze the data. Then, data were analyzed using descriptive statistics. Wilcoxon test, Chi-square, Fisher's exact test and Cramer coefficient were used to evaluate the hypotheses. The effect size of the Wilcoxon test results was estimated using the formula of r = Z / (√n).

This project was conducted in accordance with the conventions of the Helsinki Statement (Association GAotWM, 2014) and approved by the ethical committee of Mazandaran University of Medical Sciences. Consent was obtained by a family member for inclusion in the study (due to lack of consciousness in patients). No additional consumables were used which would result in increased financial burden to the patients.

## RESULTS

The majority of subjects were men (n=53, 86.9%). Subjects had an average BMI of 25.09 ± 4.32 (CI95: 25.09–26.20) and mean age of 55.18±23.43 (CI95: 49.17–61.18). The internal diameter of the endotracheal tube of most of the patients were 8 mm (n=42, 68.9%) and 7.5 (n=13, 21.3%). The place of the endotracheal tube in 32 (52.55%) and 29 patients (47.5%) was at right and left side of the mouth, respectively.

Cuff pressure of the endotracheal tube increased in all the head positions when compared to the starting position (Table 1). The greatest difference in pressure was observed when the head was in the anterior flexion and left rotation positions (p <0.001). On the other hand, results showed that increase in the cuff pressure greater than the normal range (30 cmH_2_O) was observed in all head positions (Table 2). This occurred most frequently in the anterior flexion position (50.8% cases). Also, the results of Fisher's exact test and Chi-square test showed that there was a significant difference between the position of the endotracheal tube on the right or left side of the mouth in the positions of the anterior flexion, hyperextension, left flexion, left and right rotation of the head with the cuff pressure of endotracheal tube (Table 3).

**Table 1.** Comparison of the mean endotracheal tube cuff pressure in different head positions with the mean cuff pressure in the baseline

**Head position**	**Mean (SD)**	**Mean cuff pressure in start position (SD)**	**P value**	**Effect Size**
**Anterior Flexion**	31.21(6.63)	24.57(0.86)	<0.0001	5.55(0.71)
**Hyperextension**	28.34(6.31)		<0.0001	4.08(0.52)
**Left Flexion**	28.40(4.67)		<0.0001	5.10(0.65)
**Right Flexion**	27.26(3.51)		<0.0001	5.05(0.64)
**Left Rotation**	31.26(4.66)		<0.0001	6.36(0.81)
**Right Rotation**	29.37(4.56)		<0.0001	6.44(0.82)

**Table 2.** Frequency distribution and percentage of patients in terms of endotracheal tube cuff pressure in different head positions

**Frequency (%)**	**Range of the endotracheal tube cuff pressure**		

**Head Position**	<20	20–30	>30
**Anterior Flexion**	5(8.2)	25(41.0)	31(50.8)
**Hyperextension**	5(8.2)	38(62.3)	18(29.5)
**Left Flexion**	2(3.3)	41(67.2)	18(29.5)
**Right Flexion**	0	45(73.8)	16(26.2)
**Left Rotation**	0	39(63.9)	12(19.7)
**Right Rotation**	0	49(80.3)	16(26.2)

**Table 3.** Comparison of the frequency and percentage of patients in terms of the range of endotracheal tube cuff pressure regarding its placement in the corners of the mouth and the patient's head position

**Neck Position**	**Placement of the endotracheal tube in the corner of the mouth**	**Cuff pressure of the endotracheal tube**			**P value**	**Cramer Coefficient**

		**<20**	**20–30**	**>30**		
		**Frequency**				
**Anterior Flexion**	Right Side	0	14	18	0.06	0.314
	Left Side	5	11	13		
**Anterior Flexion**	Right Side	0	24	8	0.01	0.356
	Left Side	5	14	10		
**Left Flexion**	Right Side	0	16	16	<0.001	0.492
	Left Side	2	25	2		
**Left Flexion**	Right Side	0	26	6	0.1	0.179
	Left Side	0	19	10		
**Left Rotation**	Right Side	0	26	6	0.003	0.379
	Left Side	0	13	16		
**Left Rotation**	Right Side	0	22	10	0.01	0.306
	Left Side	0	27	2		

## DISCUSSION

Our primary research focus was whether changing the patient's head and neck position could affect the cuff pressure of the endotracheal tube. Our findings suggest that during the application of each of the six head positions (anterior flexion, hypertension, left lateral flexion, right lateral flexion, left rotation and right rotation), cuff pressure increased significantly compared to the starting position. It many cases the normal range of cuff pressure was exceeded. Kako et al.(10) conducted a study on 200 patients undergoing mechanical ventilation and observed that the cuff pressure increased and decreased in 68% and 19% of the cases, respectively following changes in the head and neck position (10). Although their results are consistent with our study regarding the increased likelihood of an increase in cuff pressure as opposed to a decrease in cuff pressure, they reported a greater proportion of subjects with increased and decreased cuff pressures. This may be due to the differences in the number of samples and design study. We found that the cuff pressure of the endotracheal tube was higher than the normal range in most of the patients with anterior flexion and left rotation position. Along with this finding, another study also stated that head flexion causes the highest increase in cuff pressure (8, 10). Lizy et al. (1) investigated cuff pressure of the endotracheal tube after 16 changes in the subjects` body position. They reported that although the cuff pressure increased in all 16 positions, the cuff with the pressure of less than 20 cmH_2_O was not registered for any of the patients. They also reported that the greatest change in cuff pressure was seen in the anterior flexion position of head and right lateral position with 45 degrees head position (1). Sultan et al. (11) also stated in their review article that the change in the patient’s head position could affect the cuff pressure (11). It needs to be considered that the change of the neck and head position is not the sole factor affecting the cuff pressure. Previous studies have indicated that a change in the position and bed angle of the patients can have different effects on cuff pressure including the head of bed elevation (3, 12, 13), rotation to the left/right side, and opposing the ventilator (14). However, our findings agree with those of the above studies that position changes can lead to potentially a cuff pressure that exceeds the normal range. Therefore, we would advocate that it is necessary to monitor the cuff pressure each time the position of the head and neck is changed, especially when the head has been placed in the anterior flexion position.

We also sought to investigate which head position change causes the greatest change in cuff pressure in relation to the position of the endotracheal tube within the mouth. The results of our study showed that there was a significant relationship between each of the six head positions with placing the endotracheal tube in the corners of the patient's mouth. However, the most significant relationship was observed between the anterior flexion position and left rotation position of the head with placement of the endotracheal tube in the right side of the mouth and left side of the mouth, respectively. To our knowledge, no one has reported the effect of placing the endotracheal tube in the corner of the mouth and the head position in the changing pressure of the endotracheal tube. Soleimani and Ashrafi (15) reported that the increase in pressure associated with a change in the head and neck position and endotracheal tube placement can be due to the folding of the endotracheal tube cuff during the left/right rotation especially when moving the neck and tracheal tube traction and mechanical ventilation connections (15). This finding also emphasizes the need for monitoring and continuous adjustment of the endotracheal tube cuff pressure.

## CONCLUSION

The results of our study indicate that changes in head position of patients undergoing mechanical ventilation increase the cuff pressure of the endotracheal tube. This increase was greatest in anterior flexion position. It was also related to the endotracheal tube's position in the corners of the patient's mouth. Therefore, it is recommended to avoid unnecessary movements of the head and neck while taking care of the patient or reexamine and adjust the cuff pressure following any change in the head position. Another suggestion is the use of continuous pressure control to keep the cuff pressure within the normal and safe range.

## References

[1] Lizy C Swinnen W Labeau S Poelaert J Vogelaers D Vandewoude K Cuff pressure of endotracheal tubes after changes in body position in critically ill patients treated with mechanical ventilation. Am J Crit Care 2014; 23 (1): e1– 8. 24382623 10.4037/ajcc2014489

[2] Maddumage M Gunasekara A Priyankara WD. Endotracheal tube cuff pressure management in adult critical care units in the National Hospital of Sri Lanka; A baseline audit. Sri Lankan Journal of Anaesthesiology 2017; 25 (1).

[3] Ziyaeifard M Ferasatkish R Alizadehasl A Faritous Z Alavi SM Pouraliakbar H Effect of various patient positions on endotracheal tube cuff pressure after adult cardiac surgery. Research in Cardiovascular Medicine 2017; 6 (4): 34.

[4] Deem S Yanez D Sissons-Ross L Broeckel JA Daniel S Treggiari M. Randomized Pilot Trial of Two Modified Endotracheal Tubes To Prevent Ventilator-associated Pneumonia. Ann Am Thorac Soc 2016; 13 (1): 72– 80. 26523433 10.1513/AnnalsATS.201506-346OC PMC4722846

[5] Alharbi Z Parker K Norton G Chang D. Reduction of Endotracheal Tube Cuff Pressure During a Series of Routine Cuff Pressure Checks. Respiratory Care 2018; 63 (Suppl 10): 3024898.

[6] Akdogan O Ersoy Y Kuzucu C Gedik E Togal T Yetkin F. Assessment of the effectiveness of a ventilator associated pneumonia prevention bundle that contains endotracheal tube with subglottic drainage and cuff pressure monitorization. Braz J Infect Dis 2017; 21 (3): 276– 281. 28193455 10.1016/j.bjid.2017.01.002 PMC9427614

[7] Pesenti AM Zanella A Castagna L Scaravilli V, inventors; Universita degli Studi di Milano-Bicocca, assignee. Apparatus for controlling the pressure in an endotracheal cuff, positive-pressure ventilator for artificial ventilation of an intubated patient and method for managing secretions in an intubated patient. United States patent application US 15/573,768. 2018.

[8] Sole ML Penoyer DA Su X Jimenez E Kalita SJ Poalillo E Assessment of endotracheal cuff pressure by continuous monitoring: a pilot study. Am J Crit Care 2009; 18 (2): 133– 43. 19255103 10.4037/ajcc2009441

[9] Khalil NS Morsy WY Salama RA Sayed MS. Comparison of endotracheal cuff pressure measurements before and after nursing care in emergency patients: pilot balloon palpation. Clinical Practice 2018: 649– 53.

[10] Kako H Krishna SG Ramesh AS Merz MN Elmaraghy C Grischkan J The relationship between head and neck position and endotracheal tube intracuff pressure in the pediatric population. Paediatr Anaesth 2014; 24 (3): 316– 21. 24238105 10.1111/pan.12308

[11] Sultan P Carvalho B Rose BO Cregg R. Endotracheal tube cuff pressure monitoring: a review of the evidence. J Perioper Pract 2011; 21 (11): 379– 86. 22165491 10.1177/175045891102101103

[12] Okgun Alcan A Yavuz van Giersbergen M Dincarslan G Hepcivici Z Kaya E Uyar M. Effect of patient position on endotracheal cuff pressure in mechanically ventilated critically ill patients. Aust Crit Care 2017; 30 (5): 267– 272. 27993545 10.1016/j.aucc.2016.11.006

[13] Beccaria LM Doimo TMA Polletti NAA Barbosa TP Silva DCD Werneck AL. Tracheal cuff pressure change before and after the performance of nursing care. Rev Bras Enferm 2017; 70 (6): 1145– 1150. 29160473 10.1590/0034-7167-2016-0486

[14] Godoy AC Vieira RJ Capitani EM. Endotracheal tube cuff pressure alteration after changes in position in patients under mechanical ventilation. J Bras Pneumol 2008; 34 (5): 294– 7. 18545825 10.1590/s1806-37132008000500008

[15] Soleimani M Ashrafi R. Effect of changing position on endotracheal tube cuff pressure in patients with mechanical ventilation. Tehran University Medical Journal 2018; 76 (1): 41– 8.

